# Is Procalcitonin (PCT) a reliable biomarker for preoperative diagnosing of low grade periprosthetic joint infection? A prospective study

**DOI:** 10.1186/s12891-020-03266-6

**Published:** 2020-04-20

**Authors:** André Busch, Marcus Jäger, Harald Engler, Marcel Haversath, Charlotte Bielefeld, Stefan Landgraeber, Alexander Wegner

**Affiliations:** 1 Department of Orthopaedics and Trauma Surgery, University of Duisburg – Essen, Hufelandstr 55, 45147 Essen, Germany; 2 Institute of Medical Psychology and Behavioral Immunobiology, University Hospital Essen, University of Duisburg-Essen, Essen, Germany; 3 Department of Orthopaedics, University of Saarland, Saarbrücken, Germany

**Keywords:** Periprosthetic infection, Total joint arthroplasty, Synovial fluid procalcitonin, Complication

## Abstract

**Background:**

Since a “gold-standard” is missing, diagnosing periprosthetic joint infection (PJI) remains a challenge in orthopedic surgery. The purpose of this study was to evaluate the accuracy of serum and synovial fluid Procalcitonin (S-PCT and SF-PCT) as a diagnostic parameter and to compare it to the biomarkers recommended in the 2018 Definition of periprosthetic hip and knee infection.

**Methods:**

Between August 2018 and July 2019, a prospective cohort study was conducted in 70 patients with painful hip, shoulder and knee arthroplasty. Besides medical history, clinical and laboratory data was gathered. PJI was diagnosed based on the 2018 Definition of periprosthetic hip and knee infection. Preoperative blood and synovial joint fluid were taken for PCT measurement. S-PCT and SF-PCT levels were measured using standard quantitative PCT enzyme immunoassays.

**Results:**

Twenty three patients (33%) were classified as the PJI group and fourty seven patient (67%) as the aseptic group. The mean levels of S-PCT were significantly (*p* <  0.001) higher in the PJI group than those in the aseptic group (PJI 0.05 ± 0.21 ng/mL (0.0–1.03) vs. aseptic 0.02 ± 0.03 ng/mL (0.0–0.18)). In synovial fluid, the mean PCT values in the aseptic group were significantly higher (*p* <  0.001) than those of PJI group (PJI 2.7 ± 1.4 ng/mL (0.53–9.7) vs. aseptic 8.7 ± 2.5 ng/mL (0.25–87.9)). S- PCT, with a cut-off level of 0.5 ng/mL, had a sensitivity of 13.0% and a specificity of 91.0%.

SF-PCT, with a cut-off level of 5.0 ng/mL, had a sensitivity of 13.0% and a specificity of 52.0%.

**Conclusion:**

S-PCT and SF-PCT appeared to be no reliable biomarkers in the differential diagnosis of PJI from aseptic loosening in total joint arthroplasty.

## Background

Periprosthetic joint infection (PJI) is a severe complication after total joint arthroplasty. It is one of most common reasons for revision surgery in arthroplasty [1]. The 5-year incidence exceeds 1 % after primary arthroplasty [2]. The differentiation between aseptic and septic failure is crucial for surgical planning [3]. According to the current Consensus Definition for PJI, a minimum of two positive cultures of periprosthetic tissue or the presence of a sinus tract with evidence of communication to the joint or visualization of the implant are major criteria in diagnosis [4]. However, microbiological diagnostic is occasionally false negative or positive [5]. Conventional serum biomarkers such as white cell count (WCC) and C-reactive protein (CRP) have limited diagnostic accuracy [6, 7]. Other serum biomarkers such as Interleukin-6 (Il-6) which are often used in inflammation diagnostics reveal also shortcommings in sensitivity and specificity [8]. A wide spectrum of synovial fluid biomarkers (SF-alpha-1-Defensin, SF-CRP, SF-Il-6) have been utilized with the goal to diagnose PJI [9]. Yet, there is no “gold standard**”** for definite diagnosis of PJI [10].

Procalcitonin (PCT) has been utilized as a serum marker in detecting bacterial infection for several years [11–13]. Besides CRP serum PCT (S-PCT) seems to be the most promising biomarker to differentiate between aseptic and septic processes [14]. PCT, the precursor of calcitonin, is a 116-amino-acid protein produced by the neuroendocrine and the parafollicular cells of the thyroid [15]. In healthy patients, S-PCT level is in general very low [16], but markedly increased in severe bacterial and fungal infections [17]. It has been demonstrated that the injection of bacterial endotoxin in normal subjects induces the release of PCT systemically [18, 19]. Previous studies about S-PCT as a biomarker in diagnostic of PJI did not reveal consistent results [7, 13, 20, 21]. On the hand Glehr et al. (2013) described S-PCT as a sensitive, but less specific biomarker for detection of PJI, on the other hand Randau et al. (2014) and Bottner et al. (2007) found S-PCT to be a very specific, but a less sensitive biomarker for diagnosis of PJI [13, 21]. Until now, only one study evaluated the effectiveness of synovial fluid (SF-PCT) for diagnosis of PJI in 32 patients. Ngasoongsong et al. (2019) assessed SF-PCT as a specific, but less sensitive marker [15].

The purpose of our study was to investigate the diagnostic value of S-PCT and SF-PCT in Periprosthetic Infection in comparison to the currently recommended parameters. As the second study, we evaluate the effectiveness of SF-PCT for diagnosis of PJI. For the first time, SF-PCT is compared to the current most frequent used biomarker (SF-CRP and SF-AD-1). The hypothesis to be tested was: Due to its properties as a reliable biomarker in bacterial infection, S-PCT and SF-PCT are significantly increased in patients with PJI.

## Methods

### Study design

After approval of the institutional review board (18–8042-BO), a prospective study was performed of data gathered from Department of Orthopedics and Trauma Surgery from University of Duisburg-Essen, Germany, in patients with persisting pain [22] after hip, knee and shoulder arthroplasty.

All patients signed informed consent forms prior to being enrolled. The study was conducted in accordance with the declaration of Helsinki.

Medical history, clinical examinations, laboratory values including C-reactive protein (CRP) and joint aspiration fluid were gathered preoperatively as routine diagnostic procedures. Based on the findings of the preoperative diagnostic tests, the patients were considered as aseptic or septic according to the 2018 Definition of periprosthetic hip and knee infection [4].

The study focused on the differentiation between low-grade infects and aseptic cases. According to WAIOT definition patients without two or more signs or symptoms of local inflammation (pain, swelling, redness, warmth, function laesa) were classified as low-grade infects [23]. In order to determine the impact of renal dysfunction on serum and synovial values of PCT, serum creatinine concentrations were gathered at the time of joint puncture.

Inclusion criteria were a sufficient amount of synovial fluid for all determinations, and full clinical and laboratory data to allow for diagnosis of periprosthetic infection (PJI). Patients were further excluded, if they showed signs of early postoperative PJI (8 weeks) due to lack of reliability of synovial and serologic markers shortly after surgery [24, 25]. Metallosis, other inflammatory comorbidities (HIV, rheumatic diseases), and previous or concomitant antibiotic therapy were considered as exclusion criteria.

### Sample preparation

All patients gave their written informed consent that surplus material of their blood and synovial samples which is not needed for standard diagnostics is used for research studies.

Blood was taken from the cubital vein the day before surgery. Synovial aspiration was executed avoiding an admixture of blood with an 18-gauge needle. Synovial fluid was aseptically aliquoted into sterile tubes and centrifuged for 8 min at 4 °C with 2000 g. The synovial fluid samples were put on ice and transported within 60 min to Laboratory of Institute of Medical Psychology and Behavior Science University of Duisburg-Essen and frozen at − 80 °C.

### Determination of the levels of serum and synovial fluid biomarkers

S-PCT levels were quantified under the use of immunoassay (Centaur, Siemens, Germany) with lower limit of detection of 0.02 ng/mL (normal < 0.5 ng/mL). Serum CRP was analyzed by immune turbidimetry (Centaur, Siemens, Germany) (normal < 0.5 mg/dl). Synovial leukocyte level and percentage of polymorphic neutrophils was measured by flow cytometry with EDTA plasma (normal range, < 3000/μl and < 80%). SF-PCT levels were measured using a standard quantitative PCT enzyme immunoassay kit, according to the manufacturer’s instructions (Anti-Procalcitonin antibody ab166963, ABCAM, Cambridge,UK). Synovial alpha-1-Defensin was analyzed using a standard quantitative enzyme immunoassay kit (Human α-Defensin 1 Antibody, R&D Systems Bio-Techne, Minneapolis, USA)(cut-off level 4800 ng/mL). The results were given as standardized signal relative to a tolerance limit value (interpretation values: < 0.9 aseptic, 0.9–0.99 unspecific, ≥ 1.0 septic). Synovial CRP was analyzed under use of a quantitative enzyme-linked immunoassay (CRP ELISA (EU59131), IBL International GmbH, Hamburg, Germany) (cut-off level (> 6,9 mg / l)).

### Statistical analysis

The data were processed with the statistical software package SPSS. Basic descriptive statistics were used to analyze clinical and laboratory values. Normally distributed continuous data were shown as mean ± standard deviation (SD) and compared using student’s t-test. Non-normally distributed continuous data were shown as mean and compared using the Mann–Whitney U test. A *p* value < 0.05 was considered statistically significant. Sensitivity, specificity, area under the curve (AUC) and their 95% confidence interval (CI) for any cut-off level were calculated via receiver operating characteristic (ROC) analysis.

## Results

### Patients

From July 2018 to June 2019, 78 patients introduced themselves with persisting pain after hip, knee and shoulder arthroplasty in the consultation hour. Seventy patients could be included in the study. Three patients were excluded due to insufficient amount of synovial fluid via preoperative puncture. Two patients were excluded due to inflammatory diseases, another two due to early postoperative PJI. One patient was excluded because antibiotic therapy was already started prior to the puncture. All 78 patients who introduced themselves in consultation hour were operatively treated. In all 78 cases histological specimens were taken according to the current recommendations in the 2018 Definition of periprosthetic hip and knee infection [4].

From the 70 included patients, 47 patients were identified as having an aseptic joint effusion according to the Definition of Parvizi et al. (2018) were included into the study. The group included 27 women and 20 men with a mean age of 66 ± 12.5 (38–88) years. There were 18 knees, 27 hips and 2 shoulders. The group consisted of 45 patients with polyethylene wear debris induced osteolysis and 2 hips with corrosion of modular head-neck junction. The mean BMI (Body Mass Index) was 26.7 ± 3.1 (22–37).

In the same period, 23 patients were classified as having a PJI according to the Definition of Parvizi et al. (2018). No patient was classified as having a high-grade infect according to the WAIOT definition. The group consisted of 15 women and 8 men with a mean age of 72 ± 11.3 (47–89) years. There were 3 knees, 17 hips and 3 shoulders. The mean BMI was 27.1 ± 7.3 (19–45). In 16 aspirations joint fluid was tested positive in microbiological culture. Bacteria were identified in 16 (70%) of 23 patients of the infection group. Staphylococci were found in 11 (69%), Propioni bacteria and and Enterococci in each two (13%) and Serratia marcescens were found in one (6%). In 7 patients (29%) in the infection group with positive histologic specimens for infection, no bacteria could be isolated after 14 days incubation. The patients who were identified as having PJI were operatively revised via two- stage revision with implantation of an intermittent antimicrobial-impregnated spacer.

There were no significant differences in age (*p* = 0.32) sex (*p* = 0.53) and age at time of surgery (*p* = 0.70) between the two groups. The distribution of site of joint arthroplasty was significantly different between the two groups (*p* = 0.01) with higher rates of hip arthroplasties in the PJI group.

**Fig. 1:**
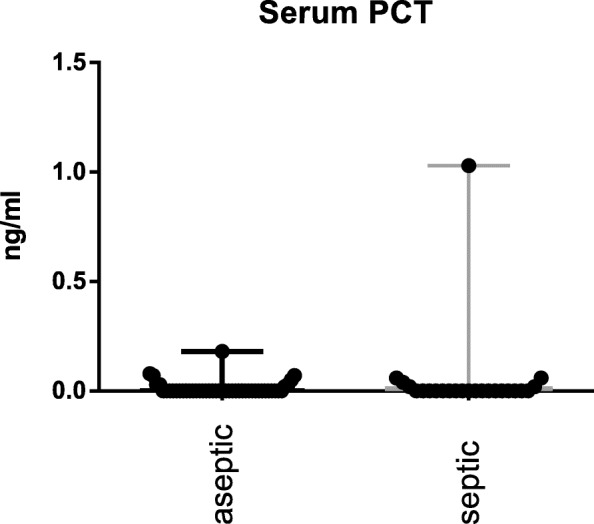
Serum PCT (S-PCT): Log-scale dot plots demonstrate the diagnostic separation of study groups

**Fig. 2:**
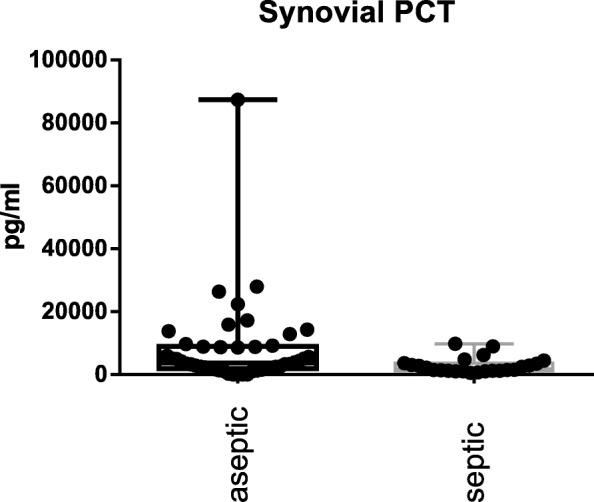
Synovial fluid PCT (SF-PCT): Log-scale dot plots demonstrate the diagnostic separation of study groups

**Fig. 3:**
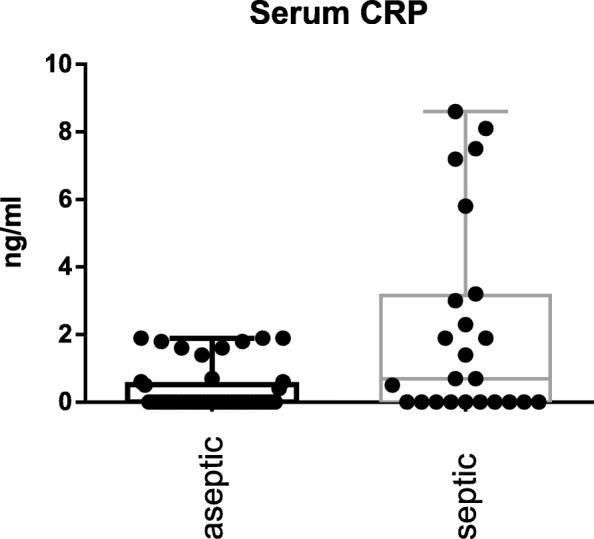
Serum CRP (S-CRP): Log-scale dot plots demonstrate the diagnostic separation of study groups

**Fig. 4:**
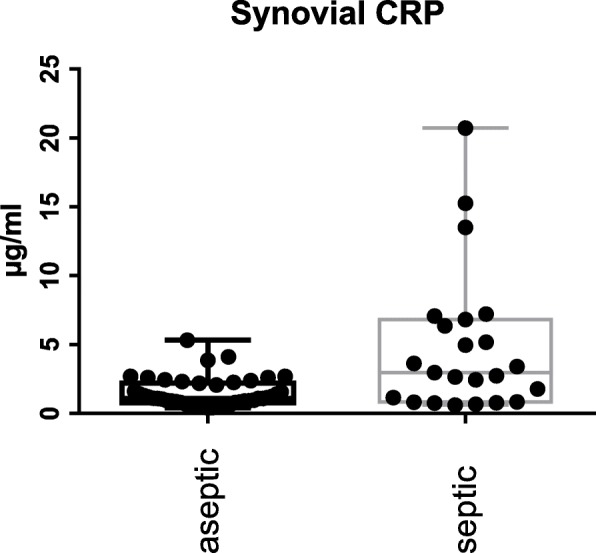
Synovial fluid CRP (SF-CRP): Log-scale dot plots demonstrate the diagnostic separation of study groups

**Fig. 5:**
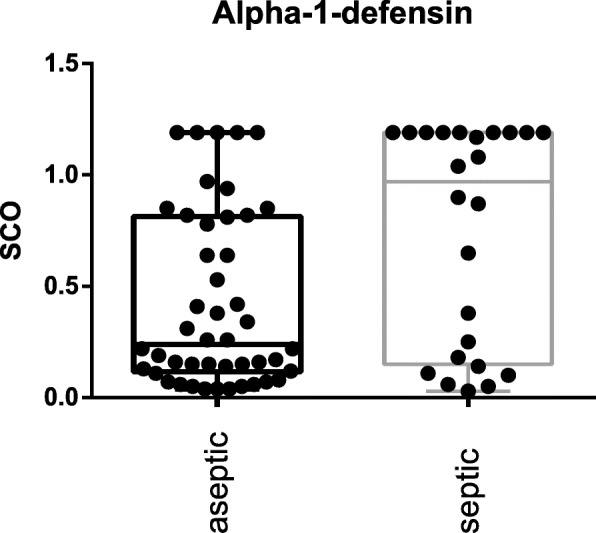
Synovial fluid Alpha-1-Defensin (SF-AD-1): Log-scale dot plots demonstrate the diagnostic separation of study groups

The S-PCT measurement was positive in 1 joint and negative in 69 (see Fig. 1). The mean S-PCT level in the PJI and aseptic groups was 0.05 ng/ml (0.00 to 1.03) and 0.02 ng/ml (0.00 to 0.18), respectively (*p* <  0.001). With a cut-off value of 0.5 ng/ml, S-PCT showed a specificity of 91% and a sensitivity of 13%. Comparing these data with the diagnosis criteria of PJI according to the Definition of Parvizi et al. (2018), it was found that the PCT-assay was false-positive in 0 and false-negative in 22 cases. The mean SF-PCT in the PJI and aseptic groups was 2.7 ng/ml (0.53 to 9.7) and 8.7 ng/ml (0.25 to 87.9), respectively (*p* <  0.001) (See Fig. 2). SF-PCT showed with a cut-off level of 5.0 ng/ml a sensitivity of 13% and a specificity of 52%. The mean serum CRP values in the PJI and aseptic groups was 2.3 mg/dl (0.0 to 8.6) and 0.35 mg/dl (0.0–1.9) respectively (*p* < 0.001) (See Fig. 3). The mean SF-CRP in the PJI and aseptic groups was 19.6 μg/ml (0.6 to 339) and 1.4 μg/ml (0.4 to 5.3), respectively (p < 0.001) (See Fig. 4). The mean synovial fluid alpha-1-defensin levels were significantly higher (*p* = 0.006) in PJI group with 3.6 μg/ml (0.2–5.7) than in aseptic group with 2.0 μg/ml (0.2–5.7). Synovial alpha-1-defensin showed a sensitivity of 52% and a specifity of 88% with a cut-off of 4,8 μg/ml (Fig. 5). The data of statistical analysis are presented in Table 1.

**Table 1:** Diagnostic accuracy of PJI (Periprosthetic Joint Infection) diagnosis using serum or synovial fluid biomarkers (CRP (C-reactive protein), AUC (Area under the curve), PCT (Procalcitonin) AD-1 (alpha-1-defensin))

Parameter	PJI (*n* = 23)	Aseptic (*n* = 47)	Cut-Off	Sensitivity	Specifity	*p*-value
Serum CRP (mg/dl)	2.3 (0.0–8.6)	0.35 (0.0–1.9)	0.5	57	81	< 0.001
Synovial CRP (μg/ml)	19.6 (0.6–339)	1.4 (0.4–5.3)	6.9	26	100	< 0.001
Serum PCT (ng/ml)	0.05 (0.0–1.03)	0.02 (0.0–0.18)	0.1	26	81	< 0.001
			0.3	17	84	
			0.5	13	91	
Synovial PCT (ng/ml)	2.7 (0.53–9.7)	8.7 (0.25–87.9)	1.0	87	0	< 0.001
			5.0	13	52	
Synovial AD-1 (μg/ml)	3.6 (0.2–5.7)	2.0 (0.2–5.7)	4.8	52	88	0.006

There were no significant differences (*p* = 0.98) in the creatinine values between the aseptic and the PJI group. Furthermore, there was no significant correlation between PCT and creatinine values (*p* = 0.68).

In Table 2, the current data are compared to the literature.

**Table 2:** Procalcitonin: Overview of sensitivity and specificity values in different studies

Author	Parameter	Cut-Off	Sensitivity	Specifity	*p*-value
Current study	Serum PCT	0.5 ng/ml	13	91	< 0.001
	Synovial fluid PCT	1.0 ng/mL	87	0	
		5.0 ng/mL	13	52	
Glehr et al. (2013) [8]	Serum PCT	0.055 ng/mL	81	54	0.038
		0.36 ng/mL	90	33	
Randau et al. (2014) [21]	Serum PCT	46 ng/mL	13	100	
Sa-Ngasoong-song P et al. (2019) [15]	Serum PCT	0.1 ng/mL	65	92	< 0.001
		0.3 ng/mL	50	100	
		0.5 ng/mL	40	100	
	Synovial fluid PCT	0.08 ng/mL	90	83	< 0.001
		0.12 ng/mL	80	92	
		0.16 ng/mL	55	91	
Bottner et al. (2007) [40]	Serum PCT	0.3 ng/mL	33	98	n.a.

## Discussion

A periprosthetic joint infection (PJI) is a serious complication after total joint replacement. Despite the existence of an international consensus for the definition of PJI, there is no “gold standard” for definite diagnosis of PJI [10]. The differentiation between aseptic and septic failure remains a key challenge in orthopedic surgery as the treatment of aseptic failure is completely different to the treatment of PJI [26].

In recent years, several studies reported on the determination of synovial and serum biomarkers for diagnosing periprosthetic infection [27–31].

CRP is a protein that is synthesized in the liver in response to acute inflammation when there are increased macrophages [32]. Several studies have endorsed the role of synovial CRP in diagnosing patients with PJI. Most studies reported that synovial CRP is a parameter with high sensitivity and specificity in diagnosing chronic periprosthetic hip infection and favorable to serum CRP [33–35]. In contrast, Tetreault et al. (2014) found no advantage to the use of synovial-fluid CRP over serum CRP in the diagnosis of PJI [36]. In our study, as expected the additional determination of synovial CRP increases the specificity, but not the sensitivity.

Alpha-Defensins are microbicidal peptides that are active against many Gram-negative and Gram-positive bacteria, fungi, and enveloped viruses [37]. Bingham et al. (2014) concluded that the sensitivity and specificity of the synovial fluid α-defensin assay is superior to other currently available clinical tests [38, 39]. However, there are also reports about low sensitivity values (64%) of synovial alpha-1-defensin [20]. In our study, synovial alpha-1-defensin was presented as very specific, but less sensitive biomarker to distinguish between aseptic and septic loosening.

The reports about PCT as a diagnostic biomarker for periprposthetic infection are inconsistent (see Table 2). Randau et al. (2014) and Bottner et al. (2007) demonstrated that S-PCT is a very specific, but a less sensitive biomarker for diagnosis of PJI [15, 21, 40]. In contrast, Glehr et al. (2013) classified S-PCT as a sensitive, but not specific biomarker for PJI detection [8]. In a systematic review and meta-analysis, Yoon et al. (2018) concluded that S-PCT is not recommended for use as a rule-out diagnostic tool for PJI [28]. Sa-Ngasoongsong et al. (2019) reported on SF-PCT as a reliable test for PJI diagnostic with high specificity and sensitivity [15]. The results of the current study show that S-PCT is a specific, but less sensitive marker for PJI diagnostic. Interestingly, the aseptic group presented significantly higher SF-PCT values than septic patients.

We could not confirm the initially described hypothesis. We believe that there is no solid evidence to recommend a single determination of S-PCT to rule out PJI. Also, the use of SF- PCT as a parameter in PJI diagnostic’s does not appear to be expedient. The lower PCT values in synovial fluid in PJI in comparison with aseptic patients may base on different reasons. Firstly, since not all patients suffering from PJI show bacteremia [41], there is no trigger for release of PCT into the blood. It is conceivable that low grade infects such as the majority of PJI do not have the virulence to trigger PCT release. Secondly, in healthy patients transient bacteremia, even after tooth brushing, is a frequent phenomenon that may induce low-grade PCT release [42–44]. Thirdly, it is well known, that in patients with chronic kidney disease PCT levels can be increased due to reduced renal elimination [45, 46]. Thus, the retention of PCT in patients with kidney diseases could results in false higher PCT values. In our cohort, we could not find any correlation between PCT and creatinine values. Finally, the penetration of PCT into the joint fluid has been infrequently studied. The penetration of PCT into synovial fluid is maybe different in each patient.

Another reason which may have an influence on the results of this study is the possible high rate of false-negatives despite the measurement of serum and synovial fluid biomarkers in addition to conventional microbiological diagnostics. Kheir et al. (2018) pointed out that surgeons should be aware of the high rate of false-negatives associated with low-virulence organisms and culture-negative cases due to low sensitivity rates. The sensitivity of the serum and synovial biomarkers appears to be related to organism type [7].

**Fig. 6:**
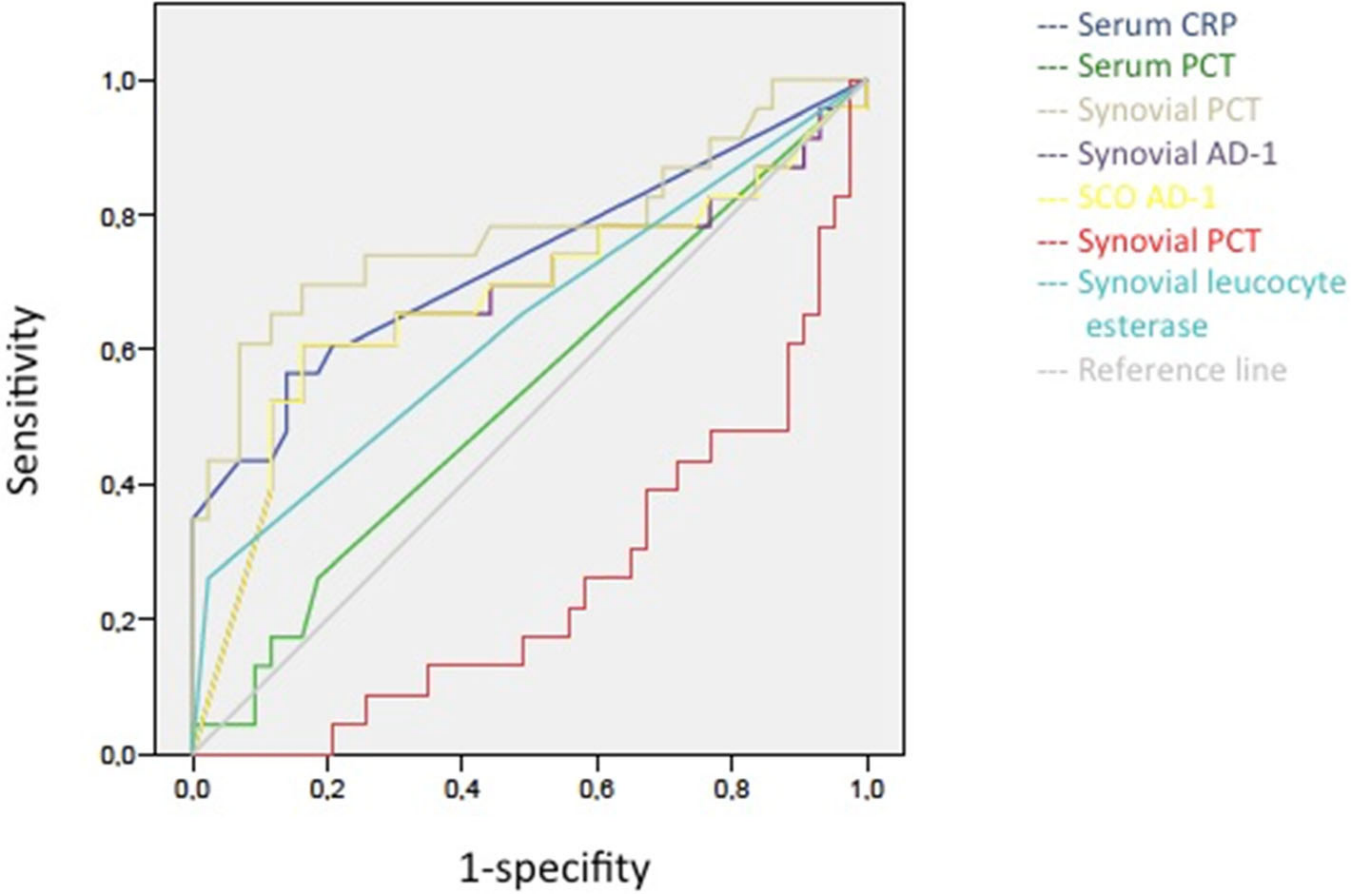
ROC (receiver operating characteristic) analysis of all parameters

In comparison to serum and synovial CRP as well as synovial AD-1, S-PCT and SF-PCT show a lack of specificity (synovial) and sensitivity (serum) and thus cannot be counted as reliable biomarkers for the differentiation between aspetic processes and PJI (see Table 3 and Fig. 6).

**Table 3:** 2018 Definition of periprosthetic hip and knee infection: (CRP (C-reactive protein), ESR (Erythrocyte sedimentation rate), LE (leucocyte esterase), PMN (polymorphonuclear leukocyte), AD-1 (alpha-1-defensin))

Major criteria (at least one of the following		Decision
Two positive cultures of the same organism		Infected
Sinus tract with evidence of the communication to the joint or visualization of the prosthesis		
**Minor criteria**	**Score**	**Decision**
Elevated serum CRP or D-Dimere	2	≥6 Infected
Elevated serum ESR	1	
Elevated synovial WBC or LE (++)	3	2–5 Possibly
Positive Alpha-Defensin	3	Infected
Elevated synovial PMN	2	
Elevated synovial CRP	1	0–1 Not Infected
**Inconclusive pre-op Score or dry tap**	**Score**	**Decision**
Preoperative Score	–	≥6 Infected
Positive Histology	3	
Positive Purulence	3	4–5 Inconclusive
Positive Single Culture	2	≤3 Not Infected

Our study has some limitations. There have been used many different wear couples. It is well known that first generation polyethylene inlays in total joint arthroplasty show higher rates of wear debris induced periprosthetic osteolysis than modern polyethylene inlays [47]. Thus, the inflammatory response with release of inflammatory biomarkers is depended on the used material. The duration from initial assessment or symptom onset to fluid collection could not be exactly assessed in all patients. Therefore, the results may be influenced by the incubation period of the germ. Furthermore, unknown factors for elevated infection parameters could have affected the outcome.

One major strengths of our study is the design as a prospective trial. To our knowledge, this is one of the first reports about PCT determination in synovial fluid. In addition, patients with chronic diseases (HIV, rheumatic diseases) which could affect the laboratory values were excluded.

## Conclusion

S-PCT and SF-PCT appeared to be no reliable alternative biomarker in the differential diagnosis of PJI from aseptic loosening in total joint arthroplasty. The frequently described high sensitivity of alpha-1-Defensin in PJI diagnosis could not be confirmed with our data.

In future studies, the detection of direct parameters for a periprosthetic infection should probably play a more prominent role .

## Data Availability

All patient-related data were collected by file research from the archives of the participating centers.

## Abbreviations

PJI: Periprosthetic joint infection
WCC: White cell count
CRP: C-reactive protein
IL-6: Interleukin-6
SF: Synovial fluid
PCT: Procalcitonin
BMI: Body mass index
SD: Standard deviation
CI: Confidence interval
HIV: Human immunodeficiency virus
